# Semi-automatic detection of honeybee brood hygiene—an example of artificial learning to facilitate ethological studies on social insects

**DOI:** 10.1093/biomethods/bpac005

**Published:** 2022-02-16

**Authors:** Philipp Batz, Andreas Ruttor, Sebastian Thiel, Jakob Wegener, Fred Zautke, Christoph Schwekendiek, Kaspar Bienefeld

**Affiliations:** 1 Adaptiv Lernende Maschinen GmbH, Hauptstraße 25, 56472 Nisterau, Germany; 2 Artificial Intelligence Group, TU Berlin, Marchstraße 23, 10587 Berlin, Germany; 3 Institute for Bee Research Hohen Neuendorf, F.-Engels-Straße 32, 16540 Hohen Neuendorf, Germany; 4 Albrecht Daniel Thaer-Institute for Agricultural and Horticultural Sciences, Humboldt University of Berlin, 10099 Berlin, Germany

**Keywords:** video analysis, behaviour detection, machine learning

## Abstract

Machine-learning techniques are shifting the boundaries of feasibility in many fields of ethological research. Here, we describe an application of machine learning to the detection/measurement of hygienic behaviour, an important breeding trait in the honey bee (*Apis mellifera*). Hygienic worker bees are able to detect and destroy diseased brood, thereby reducing the reproduction of economically important pathogens and parasites such as the Varroa mite (*Varroa destructor*). Video observation of this behaviour on infested combs has many advantages over other methods of measurement, but analysing the recorded material is extremely time-consuming. We approached this problem by combining automatic tracking of bees in the video recordings, extracting relevant features, and training a multi-layer discriminator on positive and negative examples of the behaviour of interest. Including expert knowledge into the design of the features lead to an efficient model for identifying the uninteresting parts of the video which can be safely skipped. This algorithm was then used to semiautomatically identify individual worker bees involved in the behaviour. Application of the machine-learning method allowed to save 70% of the time required for manual analysis, and substantially increased the number of cell openings correctly identified. It thereby turns video-observation of individual cell opening events into an economically competitive method for selecting potentially resistant bees. This method presents an example of how machine learning can be used to boost ethological research, and how it can generate new knowledge by explaining the learned decision rule in form of meaningful parameters.

## Introduction

Social insects are dominant species in many ecosystems. They are thought to represent more than 50% of global insect biomass, although they only account for approximately 2% of species diversity [1]. The key to this success is cooperation, based on division of labour and the exchange of materials and information (reviewed, e.g. in Refs [2, 3]). The study of the self-organization and decision-making in insect societies has not only been an important topic of ethological and sociobiological research. ‘Swarm intelligence’ has also inspired new approaches to optimizing algorithms in fields as diverse as learning theory, chemical engineering and business management [4–7]. In recent years, this debt is beginning to be paid back, through the development of computer-based methods of analysis for complex insect behaviours and behavioural networks. Decision-making in insect societies is almost always a decentralized process, depending on multiple interactions and events of stimulus perception (reviewed in Refs [2, 8]). A particularly fruitful approach has been the computer-based recognition of behavioural patterns derived from video footage: algorithms can find uncapped cells [9] or interactions of bees [10–14] in videos of a bee comb, they analyse the nursing behaviour of bees [15], the movements of ants [16–18] and the task allocation of social insects [19, 20]. Often animals are first tracked manually [21] or automatically [10, 17, 19, 22] before the recorded location and orientation data are evaluated by data analysis algorithms [10, 13, 14, 16, 17, 22] and machine-learning methods [11, 18], but extracting more features from the video [11, 12, 15, 20, 23] can improve the discrimination of different behaviours and/or different individuals.

Here, we show how a similar approach can be used successfully for an applied purpose. We describe the example of a machine learning-based method to assess an important resistance mechanism of honeybee (*Apis mellifera*) workers against the parasitic mite, *Varroa destructor*. Pollination by honeybees is crucial to many ecosystems as well as the agricultural production of many crops [24–27]. Varroosis is one of the most important causes of colony losses nearly worldwide [28–30]. The mite reproduces inside capped honeybee brood. Hygienic behaviour, that is the detection, uncapping and removal of diseased brood, can efficiently reduce the reproduction of the parasite [31–33]. It is also seen as an important element of social immunity with regard to other economically important bee diseases such as American foulbrood, chalkbrood and tropilaelapidosis [34–36]. Over the last decades, it therefore has become an important breeding trait (see e.g. [37–43]). The most widespread bioassays for measuring brood hygiene involve damaging brood by freezing or transpiercing, placing it back into its colony of origin and visually assessing removal by worker bees after a given period of time (reviewed in Ref. [44]). These assays are relatively easy to perform and have been used successfully. However, the correlation between their results and the natural stimuli emanating from infested/infected brood is not always strong [45]. We have recently described an assay for honeybee brood hygiene that has the advantage of involving the natural stimulus, infested brood and also allows for the identification of the individual worker bees involved in the two components of hygiene, that is detection and removal [46]. This assay uses video observations of tagged bees on an artificially infested comb. It also enables the detection of incomplete hygiene, that is openings of brood caps that are later closed by other worker bees. This so-called ‘re-capping’ can mask the presence of hygienic bees when hygiene is measured using the traditional assays. In the case of Varroa-infestation, recapping itself is thought to contribute to a reduced fertility and fecundity of the female mites [31]. Manual analysis of the video footage turned out to be very time-consuming, so that an algorithm for automatically detecting uncapped cells was developed [9]. However, bees often occlude the cells and thereby delay the detection in practise [47].

The aim of the present study was therefore to develop and validate a machine-learning solution to automate the detection of the hygienic behaviour itself. For this purpose, we have to select an appropriate learning algorithm which can be trained on a limited amount of training data and optimally balances detection quality with computation speed. In recent years, deep learning algorithms have become the state of the art as a powerful integrative method for learning both the model and the feature vector based on the training data alone. While they deliver impressive results in a number of image and video classification tasks, the number of examples necessary for fully automatized learning from video data, which is typically required for deep learning algorithms, is often hard or impossible to obtain [48–50]. One way to approach situations with limited training data is to integrate prior knowledge of domain experts into the learning process. This allows to reduce the complexity of the problem in such a way that the algorithm is able to learn and generalize from the available examples [51, 52]. This is an active research topic also in non-deep learning [53, 54]. Integration in conventional machine learning is typically achieved through appropriate regularization with modified loss functions or adaptions of prior probabilities in a Bayesian context. In our case, we use the biological expert knowledge in order to manually design an informative low dimensional feature space. Then a Bayesian model is used to incorporate this prior information into the learning process: The nonparametric Gaussian processes (GPs) not only allow us to efficiently learn nonlinear relationships from few training data points, but also offer a straightforward way to automatically infer the influence of the explainable features by hyperparameter learning, which is an advantage over non-Bayesian machine-learning methods like the popular support vector machine [55]. The interpretability of importance and relationship of the individual feature components is an advantage compared with often used black-box methods, for example generic deep neural networks, especially for modelling more complex relationships like interactions and social behaviour [54, 56]. For our application, we learn an ensemble of GPs on different time frames and use the posterior predictions in order to train a final detection discriminator. Here, we choose a boosting algorithm with a mean-squared error loss, which despite its simplicity gives very good results in practice.

## Materials and methods

### Video recordings of hygienic behaviour

The protocol used is described in detail in Ref. [46]. Around 2,000 worker bees are individually tagged with numbered plastic tags. On a comb of freshly capped brood, 70 cells are artificially infested by inserting one female *Varroa destructor*-mite through a small cut in the cell cap. The cells are closed again with a brush. The comb is placed inside a wire mesh cage, one side of which is replaced with a glass pane. The cage with the comb is then introduced into a full-sized colony for climatization. The bees on the comb are observed for 2–6 days through an opening in the side of the hive under near infrared illumination (650 nm), at a resolution of 2432 × 864 pixels and a framerate of 10/s. The volume of data produced for each round is of the order of 10–17 TB. The desired output from analysing these data is

- for each of the 50–70 infested cells on the comb, an assessment of whether it is uncapped during the experiment or not,
- in case it is uncapped, the identity of the bee who performed the uncapping, as well as of the first bee continuing it,
- the time of occurrence of these events (position in the video).

In the past, the identity of the bees opening infested cells (the bee initiating the uncapping as well as the first two bees continuing this task) was determined by human observers, using the following method:

- determine which of the infested cells have been opened within the course of the experiment, by checking on the last frames of the recordings, as well as at irregular intervals in earlier positions of the video,
- for each emptied cell, jump backwards and forwards in the video file to identify the moment when the first small opening occurs in the cap,
- identify the bee producing the first hole and continue viewing the recording from this position at about two times the natural speed until the first ‘continuer’ has been found.

### Development of the machine-learning algorithm for detection of hygienic behaviour

Modelwise, detecting honeybee brood hygiene in video recordings is an instance of rare event detection in large, high-dimensional data sets. Models of this type are typically characterized by three main challenges [57, 58]:

- Learning a model on the basis of a very limited amount of rare event observations.
- Reliably detecting the rare events in question while keeping the amount of false positives to a minimum.
- Analysing data fast enough for real-world usage.

In order to meet these objectives, we first define a low dimensional feature set, which maps the input data into a low dimensional feature space, while retaining the relevant information for discriminating the rare events form all other events. The specific features used in our application are manually designed based on biological expert knowledge about brood hygiene behaviour of honey bees.

Once the feature vector is calculated, we analyse the data in a three step process:

1. *Selection step*: In the first step, we use a hidden Markov chain as a fast preselection tool in order to filter out all data which clearly do not pertain to an opening process.
2. *Analysis step*: Next, the remaining data are separately analysed by GPs over different time windows. GPs are computationally more costly than hidden Markov chains, but allow for complex nonlinear data modelling.
3. *Decision step*: The final decision for a cell opening is computed by an optimal combination of the analysis step results determined by a boosting algorithm.

We will start by giving a short overview about the tracking algorithms, then take a closer look at the specific feature vector and data analysis components used and finally describe the labelling process.

### Tracking

Collecting the input data for learning the relevant underlying behavioural pattern presupposes a robust and efficient identification of all bees in a given frame as well as their tracking in consecutive frames. In order to identify individual bees, we use the fact that the numbered tags have a standardized size and can be clearly identified due to their light colour against the dark background of the bee’s thorax. Specifically, we employ binary thresholding in order to highlight the relevant areas and subsequently detect tag sized connected regions in the binary image [59, 60]. This approach turns out to be very robust even under slight variations of lighting situations and produces almost no false positives. Once the locations of the tags are identified, we track the corresponding bees over time by using a fast approximate nearest neighbour algorithm, which matches the detected connected regions over consecutive frames [61]. Although the algorithm occasionally looses bees moving at fast speed, in practice the tracking has a high accuracy around the relevant cell opening events, since these situations are characterized by bees moving at low velocity and exhibiting small thorax motion. For the purpose of gathering relevant head motion information, we first have to identify the head location for each corresponding tag. We accomplish this by searching for the eyes, which are characterized by their dark colour, using inverse binary thresholding in a circular shaped region centred around the tag and with a diameter defined by the distance between a bee’s head and its tag. Once the eyes are identified, we employ an optical flow algorithm for the detection of moving connected fields between consecutive frames [59], where the head motions are recorded as either left or right movements relative to the bee’s thorax motion.

In order to gather more concrete data for our cell opening detection task, we additionally connect motion information of bees with location information on cells. Here, the underlying idea is that beginner cell openings are characterized not only by a bee staying a certain period of time at the cell but more precisely staying at a very specific point on the cell cap during the opening process. We model this behaviour by dividing each cell into a regular grid of 24 squares, where for each frame we record which of the squares are (partially) covered by a bee’s head. In cases of cell opening events, the data yield characteristic distributions of grid square coverings over the relevant time frame. The collected data for a detected bee are only stored if its tag is currently located inside a circular region around one of the Varroa infested cells, otherwise the information is discarded. For cases where two or more bees are detected around a particular cell at a given time frame, the values are added.

It is important to emphasize that the input data are analysed with respect to the individual Varroa infested cell in questions and not with respect to individual bees. Therefore, only the subset of recorded data relevant to the problem will be stored and inspected. Also, this approach is robust against a temporary loss of a bee from the tracking process (i.e. due to full or partial occlusion of its tag by other bees), since after recovery the data can easily be matched to the correct cell without expensive tag analysis. We did not include an automatic tag recognition in our application, since in our semi-automatic system all potentially interesting events detected by the algorithm are inspected by a human expert, so for each identified cell opening event the corresponding numbered tag can directly be read off without extra effort.

**Figure 1:**
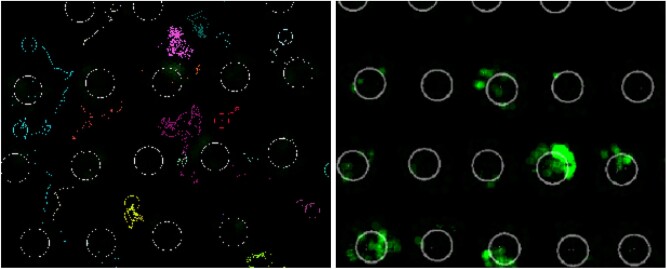
The plot on the left displays detected tracking data over time *t*. Here, the coloured trajectories denote the thorax movements of individual bees with the coloured circle representing the current position of the tag, while white circles denote the infested cells. The plot on the right shows the head motion intensity at the infested cells, where green areas denote locations of high activity.

Figure 1 shows an example of thorax and head movement input data collected by the tracking algorithm on the left and spatial head motions on the cell cap on the right.

### Feature vector

**Figure 2:**
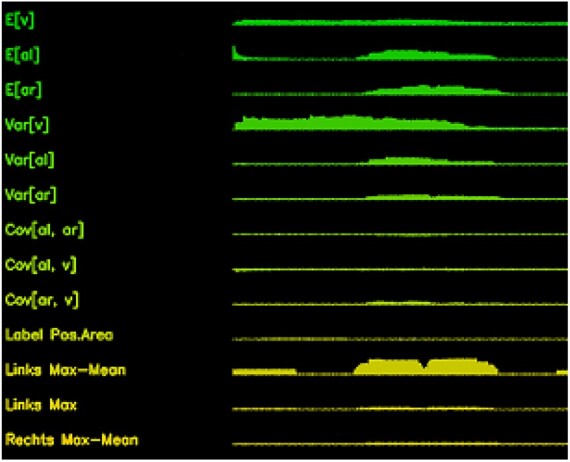
Plot of a feature vector time series snippet of a cell with the first 13 features as defined in Table 1. From this figure, the feature vector at some specific time frame can be found visually by ‘vertically cutting’ the plot and reading off the corresponding feature values.

The feature vector contains different statistics computed from the collected data of each infested cell over a fixed time horizon. For each new video frame, the features are recomputed according to a sliding window approach, where the data of the current frame are included in the feature computation and data from the oldest frame are discarded if outside of the corresponding time window. Specifically, we use three different sets of feature vectors with time windows of 1, 2 and 5 s, respectively. Here, the underlying idea is that different time resolutions each contribute different informational aspects about the event in question, which in combination should lead to better detection results. Since individual head shaking movements which indicate an uncapping event are shorter than the shortest of our time windows, we can reasonably expect to capture these basic ‘building blocks’ in the feature vector. On the other hand, the chosen time windows are typically relatively short compared with the duration of a complete uncapping event and therefore allow for extracting multiple observations from one event. The 16 different features used are given in Table 1. In Figure 2, an example of feature collection over time by the tracking algorithm at an infested cell is shown.

**Table 1:** The table of the 16 features used with short description

1	E[v]	Mean velocity (in time window)
2	E[al]	Mean head activity left
3	E[ar]	Mean head activity right
4	Var[v]	Variance velocity
5	Var[al]	Variance head activity left
6	Var[ar]	Variance head activity right
7	Cov[al, ar]	Covariance head activity left and right
8	Cov[al, v]	Covariance head activity left and velocity
9	Cov[ar, v]	Covariance head activity right and velocity
10	A[*x*, *y*]	Rectangular area of motion covered in x and y positions
11	max(cl) − E[cl]	Difference between max and mean left head activity in cell grid
12	max(cl)	Max left head activity in cell grid
13	max(cr) − E[cr]	Difference between max and mean right head activity in cell grid
14	max(cr)	Max right head activity in cell grid
15	En[c]	Mean overall head motion entropy of cell grid
16	min(*n*, tw)	Length of current trajectory w.r.t. time window

Specifically, the features include thorax statistics (features 1, 4, and 10), head motion statistics (features 2,3,5,6,11–15) and covariance statistics between thorax and head (features 7–9). While features 2, 3, 5 and 6 provide simple head motion intensity statistics, features 11–15 contain information on spatially resolved head motion intensity on the cell grid area, capturing high activity on locally confined subregions of the cell cap. Finally, feature 16 indicates the time window length of the current feature vector data, denoting if the time window of the data is shorter than the allotted time horizon for instance due to loss of tracking.

For the following discussion, we assume that we have *N* observations $D=\left\{\left(x_{n},y_{n}\right)\right\}$ with n=1,…,N, where $x_{n}$ denotes the *M* dimensional feature input vector for the *n*-th observation and *y_n_* its corresponding label. Note that *y_n_* = *z* can take on two values z=±1.

### Hidden Markov chain

We first analyse the activity of the bees at each cell using a hidden Markov chain [57]. The hidden state $y_{i,t}$ at time point *t* is binary, either a bee is opening the cell *i* (active state $y_{i,t}=1$) or not (inactive state $y_{i,t}=-1$). The features are treated as observations $x_{i,j,t}$ with j∈{1,…,M} of the hidden state $y_{i,t}$. We use a simple ‘transition model’,

(1) $$P(y_{i,t+1}=1|y_{i,t}=-1)=\frac{\alpha }{\tau (1-\alpha )},$$

(2) $$P(y_{i,t+1}=-1|y_{i,t}=1)=\frac{1}{\tau },$$

which assumes that opening a cell takes *τ* time steps on average and that without observations the stationary probability to find any activity is *α*. In our programme, we use *τ* = 300 frames (≡30 s) and $\alpha =10^{-3}$. Note that these prior assumptions can be rough estimates only used to regularize the model, since normally the information obtained from the observations will dominate the results [62].

The observation model is learned from positive and negative examples, where the value of the hidden state $y_{i,t}$ is provided by the user. We assume that all features are mutually independent of each other and are drawn from a (possibly truncated) Gaussian distribution. While the features describing different aspects of the same motion are certainly not independent, this is neglected in the model in order to speed up inference and to reduce the number of parameters to be learned. The parameters $\mu_{j,z}$ (mean) and $\sigma_{j,z}$ (standard deviation) of each Gaussian distribution are calculated from the empirical moments of feature *j* conditioned on positive (*z* = 1) or negative (*z* = −1) examples. For features with only positive values, for example the variance of the velocity (feature 4), the Gaussian distribution is truncated to this range and renormalized.

After training the hidden state $y_{i,t}$ of a cell *i* at time step *t* can be predicted from observations by filtering. The goal here is to calculate the probability $p_{i,t}=P(y_{i,t}=1|D_{\leq t})$ for $y_{i,t}=1$ given all observed features $D_{\leq t}$ up to time *t*. At the start of the video, *t* = 0, the probabilities for all cells are set to $p_{i,0}=\alpha$. Then, the predictions $p_{i,t}$ for time steps *t* > 0 are calculated iteratively using the filter equations of the hidden Markov model.

If $p_{i,t}$ reaches at least a threshold β=0.25, this indicates significant activity at position (*i*, *t*) which is analysed further using the GP model described below. That way it is possible to disregard a large part of the video without interesting activity quickly. But due to the simplified assumption of independence between the features, it is not possible to find cell openings (without producing a lot of false positives) using this filter alone. Hence, we further analyse the remaining data with the help of GP methods.

### Gaussian processes

A GP is a nonparametric Bayesian inference tool for regression and classification tasks [55]. It has been successfully applied to problems from a variety of different backgrounds. Due to its nonlinearity, the GP is also able to capture more complex functional relationships between its input and output variables.

Although detecting cell openings with labelled data ±1 naturally lends itself to a GP classification model, for computational reasons we decided to use GP regression instead, since due to its analytical tractability GP regression predictions can be computed significantly faster than their GP classification counterparts. Moreover, carrying out regression directly on the labels has been shown to give results comparable to classification for GP estimators applied in a frequentist context [63].

In a regression framework, we assume that a label *y* is generated by the underlying functional mapping f(x) from a *M* dimensional input space to an one-dimensional output space plus observation Gaussian error term with zero mean and variance $\sigma^{2}$. Using vector notation for labels $y={\left(y_{1},\ldots ,y_{N}\right)}^{T}$ and function values $f={\left(f(x_{1}),\ldots ,f(x_{N})\right)}^{T}$ and defining **I** as identity matrix, the likelihood for the complete observations has the form $p(y|f)=N(y|f,\sigma^{2}I)$.

As prior function p(f) over the set of functions **f** we use a GP, which for any finite collection of function variables $f=(f(x_{1}),\ldots ,f(x_{N}))$ defines a Gaussian distributed joint probability with mean zero and covariance **K**: p(f)=N(f|0,K). The covariance matrix **K** defines the pairwise covariance of the input locations $X=(x_{1},\ldots ,x_{N})$. Its components, covariances between **x** and x′, are given by the ‘kernel’ function k(x,x′).

For our application we use the ‘radial basis function’ (RBF) kernel for ‘automatic relevance determination’ (ARD) defined as

(3) $$k(x,x\prime )=\theta \;\text{exp}\;\left\{-\sum_{i=1}^{D}\frac{{\left(x_{i}-x_{i}^{\prime }\right)}^{2}}{2l_{i}^{2}}\right\},$$

which has a characteristic length scale *l_i_* for each feature *i*. Predictions in this framework can be calculated quickly using linear algebra [55].

Another benefit of the GP framework is that all the free parameters (usually called ‘hyperparameters’) $\Theta =(\theta ,l_{1},\ldots ,l_{M},\sigma )$ of the model can be learned from the data in a straightforward way by optimizing the marginal probability p(y|Θ) with respect to Θ. In case of the kernel (3), this optimization includes ARD as the optimal values of the length scales $l_{i}^{*}$ indicate the relative importance of each feature *x_i_* for the prediction of *y*. Using ARD can help to discard data dimensions which have little influence on the result and more generally gain an insight into how much each dimension contributes to the actual prediction.

For our application, we independently train three different GPs ${\text{GP}}_{1}{,\text{GP}}_{2}{,\text{GP}}_{3}$ on the three different time windows and use these for prediction. In a last step, we use a boosting algorithm in order to combine these predictors to arrive at a final classification decision.

### Boosting

Techniques that combine different classification or prediction models in order to improve the overall performance relative to each individual member are known as ‘boosting methods’ in the machine-learning community [62]:

(4) $$E_{\text{Final}}(y|x_{i})=\sum_{i=1}^{3}\alpha_{i}E_{{\text{GP}}_{i}}(y|x_{i}).$$

The values of the weights $\alpha_{1},\alpha_{2},\alpha_{3}$ are determined by minimizing a suitable loss function on the training data.

Since the predictions of the GPs ${\text{GP}}_{1}{,\text{GP}}_{2}{,\text{GP}}_{3}$ are regression outputs, we use the ‘mean-squared loss’ function between the label and prediction on the training data set D:

(5) $$L_{MSE}(x_{i})=\frac{1}{n}\sum_{i=1}^{n}{\left(y_{i}-E_{\text{Final}}(y_{i}|x_{i})\right)}^{2}.$$

Further details can be found in Ref. [62]. We then classify data $x_{i,t}$ as cell opening, if the trained booster satisfies $F_{B}(x_{i,t})>c_{{\text{Boost}}_{\text{thresh}}}$. For a discussion of different boosting algorithms and different loss functions, which can also be customized for rare-event applications, see Refs [48, 64].

### Labelling

Now, training the machine-learning classifier requires data of cell opening examples in form of feature vectors as defined above. Since in our case the cell opening examples were provided as a list of time stamps for each of the 50 marked cells in the bee hive video, we implemented a graphical user interface in order to facilitate the labelling process. It allows the user to load a video of the bee comb and jump to an arbitrary time point, generating a training example with corresponding feature vectors for the different time windows by simply clicking on a specific bee at one of the cells. The user can then decide if the recorded example counts as an example of a cell opening or an example which is not directly related to a cell opening. For the negatively labelled examples, we used bees at randomly chosen cells and time points, which nonetheless exhibited behavioural patterns not too dissimilar from cell opening motions. Thus, providing informative negative examples allows the trained classifier to decide more reliably in ambiguous cases.

### User interface for semi-automatic analysis of behaviour

Already early in the process of validation, the number of false positives, that is peaks which could not be attributed to cell uncapping behaviour, proved to be relatively high. The most frequent source of these unqualified peaks was trophallactic behaviour, that is the exchange of food between bees, which is accompanied by turnings of the head similar to those used for training of our discriminator. Additionally, the signal-to-noise ratio varied from cell to cell, so that it was difficult to decide which threshold should be applied to the height of the peaks for fully automatic classification. This lack of specificity prompted us to renounce to the idea of a fully automatic detection of uncapping and instead opt for a computer-aided, semi-automatic system.

**Figure 3:**
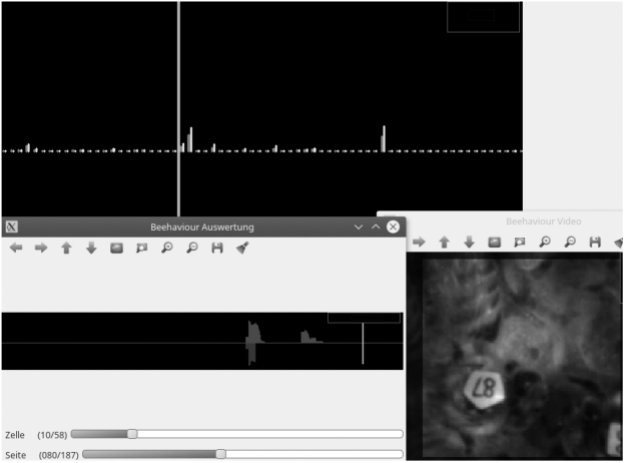
Screenshot of the user interface for verifying the results of automatic detection. Upper part: diagram of the discriminator score (*y*-axis) with time on the *x*-axis. Lower left part: zoomed segment of the diagram. Lower right part: corresponding section of the video showing the cell cap in question.

The software analyses the probability of the learned event (in our case, head nodding and shaking associated with cell uncapping) separately for different areas of the picture (in our case, different infested brood cells). The user interface displays these probabilities over time, for an area that can be chosen by the user, in the form of a diagram (Fig. 3).

The user designates an area where the probability for uncapping activity is high with the mouse arrow, upon which an extract of the original video sequence, corresponding to the time and screen area chosen, is displayed, enabling the user to quickly verify the correctness of the accession. Confirmed cases of the behaviour are then directly written into a text file using the ‘comma separated values’ (CSV) format, which is readable by practically all spreadsheet applications and data processing software.

## Results

### Training of the discriminator

As training data we used video material of worker bees on a brood comb with 50 marked (artificially mite-infested) cells, from which we collected *N* = 2049 observations. Each observation is represented by a 16-dimensional feature vector for each of the three time windows. Out of the 2049 observations, 1060 are from cell opening events and the remaining 989 observations from non-cell opening events. The fact that we were able to collect multiple cell opening observations per marked and opened cell is due to two circumstances: most cell openings are significantly longer than our defined time windows of up to 5 s and many recorded cell opening observations are partially overlapping. With the help of the label interface, we were able to generate the training set in around 5 h.

### Performance

The software processes about 8 frames per second using 8 threads on a machine with Intel i7 processor. Therefore, its run time is approximately 20% slower than real time for videos recorded with 10 frames per second.

### Feature discriminatory power

**Figure 4:**
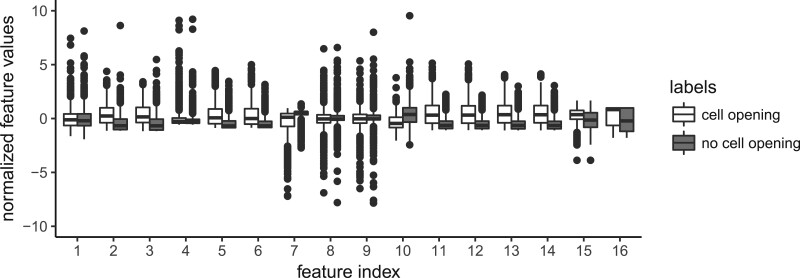
Comparison of the empirical distributions for cell openings (white box plots) and non-cell openings (gray box plots), calculated on the data for the longest time window (5 s), for each of the 16 features. For better comparison, we normalized the data set for each feature to mean zero and standard deviation one before splitting it according to the labels.

In order to gain insight into the discriminatory power of the individual features used, we compared the corresponding empirical distributions for the cell opening labels and non-cell opening labels for each feature. Figure 4 shows the box plots for the longest (5 s) time window. For many features, a notable difference in the corresponding distributions is visible both in terms of their means as well as variances, indicating that the particular feature contains relevant information for discriminating a cell opening event from the remaining bee activity near this cell. An overview of the sixteen features is given in Table 1.

### Feature influence

Next, we considered the feature influence in the three GPs by inspecting the corresponding length scales learned by the ARD. Specifically we look at (normalized) weights defined as the empirical standard deviation divided by the learned length scale *l_m_* for each feature. Large weights denote a large influence of the corresponding feature in the kernel matrix and consequently in the computation of the prediction. Table 2 shows the three most predictive features for each of the three time windows: for all three vectors, the three most important features are a combination of thorax and head information. In the two shorter time windows, the GP selects movement information both at the cell itself (rectangular area of motion) and on the cell grid (head motion entropy) at higher resolution as discriminating features. For the longest time window, different variance statistics are dominating, although here the weights are more evenly distributed (smallest weight 0.03) than in the two shorter vectors (smallest weight < 0.01).

**Table 2:** For each of the three different time windows (columns), the three most influential features in the GP regression are shown with their corresponding weight given in parenthesis

1 s		2 s		5 s	
Head motion entropy	(0.24)	Rectangular area of motion	(0.16)	Variance of velocity	(0.17)
Rectangular area of motion	(0.12)	Variance of velocity	(0.13)	Right head activity variance	(0.09)
Left head activity mean	(0.10)	Head motion entropy	(0.11)	Left head activity variance	(0.09)

*Notes*: Note that the sum of weights for each complete feature vector is one. The automatic feature weight computation is based on the ARD method.

### Feature design

Note that in our algorithm, the user can use any features, either off-the-shelf or specifically designed for the particular application, and subsequently evaluate their specific influence in the GP regression just by inspecting the corresponding learned weight as seen in Table 2. So one straightforward way to use this approach in practice is to start with many different features, learn the normalized GP feature weights with the ARD method and subsequently remove all features, whose weight is less than some small threshold value.

### Time interval influence

After computation of the GPs for the three time windows, we combine these learners by boosting in order to get the final classification. The influence of the different time windows on this decision can be read directly from the learned booster weights for the corresponding GPs. For our data, the weights (which sum up to one) are 0.21 for the 1 s time window, 0.22 for 2 s and 0.57 for 5 s. The GP for the 5 s time window is clearly the dominating component in the booster, although the other two GPs contribute a non-negligible proportion to the final decision.

### Learning curve

**Figure 5:**
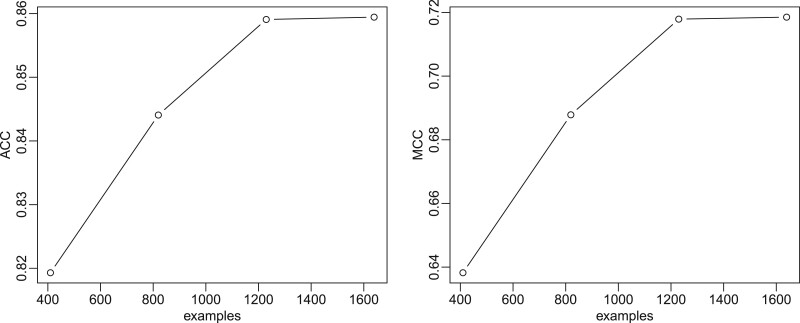
Accuracy (left) and MCC (right) learning curves of the classifier. In both figures, the dots denote the observed values, the lines are linear interpolations between observations.

We also analysed the influence of the number of examples on the quality of the prediction. For this purpose, the collected examples were split into 5 groups and all 75 combinations of one group as test set and one to four other groups as training set were tried out in a procedure similar to five-fold cross-validation [62]. Figure 5 shows two different measures as a function of the number of examples in the training set. The graph on the left displays the accuracy while the graph on the right shows the Matthew correlation coefficient (MCC). The MCC is often used in machine learning as a measure in rare event detection to better take different class sizes into account. However, since we trained our algorithm on a roughly equal number of positive and negative training data, the accuracy and MCC display the same characteristic with respect to the number of training data. Since both curves level off after 1200 examples, we decided to stop collecting further data for the training process.

## Validation

### Validation of the discriminator

**Figure 6:**
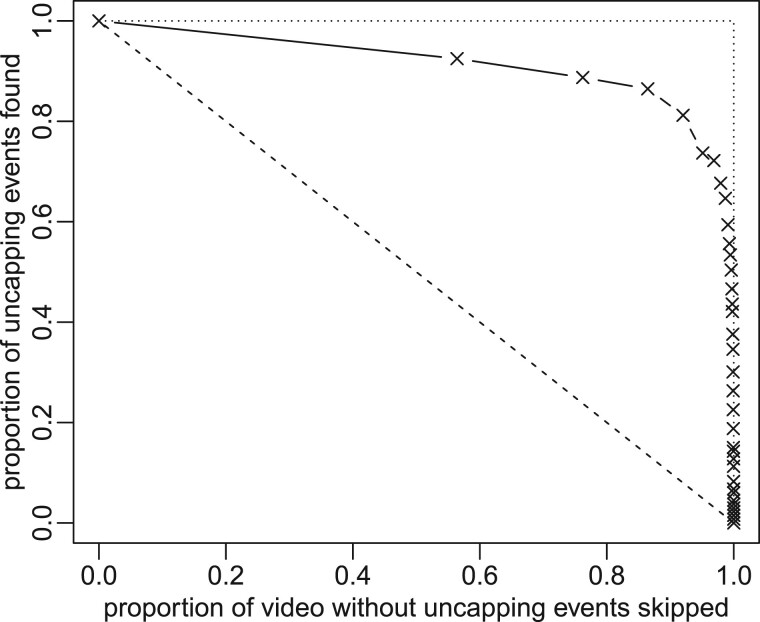
The ROC curve shows the proportion of uncapping-events that can still be detected when discarding different proportions of the original video material, judged by the algorithm as being of lesser relevance. The crosses represent combinations which were evaluated, the solid lines give linear interpolations between adjacent evaluations. Random guessing would lead to the dashed line, while a perfect classifier would reach the dotted line.

In order to optimize the ratio between time invested for the checking of results and the completeness of detections, we tested different probability thresholds of the nodding-and-shaking-behaviour. The ground truth consisted of two time intervals for each infested cell in the video denoting the first (beginner bee) and second (helper bee) uncapping behaviour found by manual inspection. We then divided the video into intervals of 450 s and calculated the mean probability score for each time interval and cell. These values are displayed in the overview window of the software. For each threshold, we then counted sections with uncapping behaviour and mean score over the threshold (‘true positives’: TP), sections with uncapping behaviour and mean score under the threshold (‘false negatives’: FN), sections without uncapping behaviour and mean score over the threshold (‘false positives’: FP) and sections without uncapping behaviour and mean score under the threshold (‘true negatives’: TN). As we did not have manually obtained information about further uncapping actions, the counting at each cell was stopped after the first helper bee. Figure 6 shows the proportion of uncapping events found, the sensitivity, as a function of the proportion of the video (not containing uncapping events) skipped, the specificity. In the literature, this is known as the ‘receiver operating characteristic’ curve [65].

The results clearly show that using this simple decision rule a large part of the video can be skipped without missing many uncapping events. Often the area under the curve is used to score the performance of classification algorithms [65]. While random guessing would yield the dashed curve and ${\text{AUC}}_{\text{random}}=0.50$, the best value reachable in this experiment is ${\text{AUC}}_{\text{best}}=1.00$ (dotted line). Our algorithm with a simple threshold for the booster output achieves AUC≈0.91, which is not far from the ideal case [65].

### Validation of the computer-aided method of detection

The semi-automatic method described above, involving a computer-based detection of likely events that are then checked by a trained human observer, was validated by twice analysing three rounds of the experiment, once by an experienced human observer (‘manual analysis’) and once by largely untrained personnel using the ‘semi-automatic analysis’. The total number of uncapping events detected by either one or both methods was divided up as follows:

- detected by both methods, same initiating bee identified, acting at the same time;
- detected only by either of the two methods;
- detected by both methods, but at an earlier stage by one than by the other (with the effect that a bee identified by one method as a mere ‘continuer’ of uncapping was classified as the initiator by the other).

**Figure 7:**
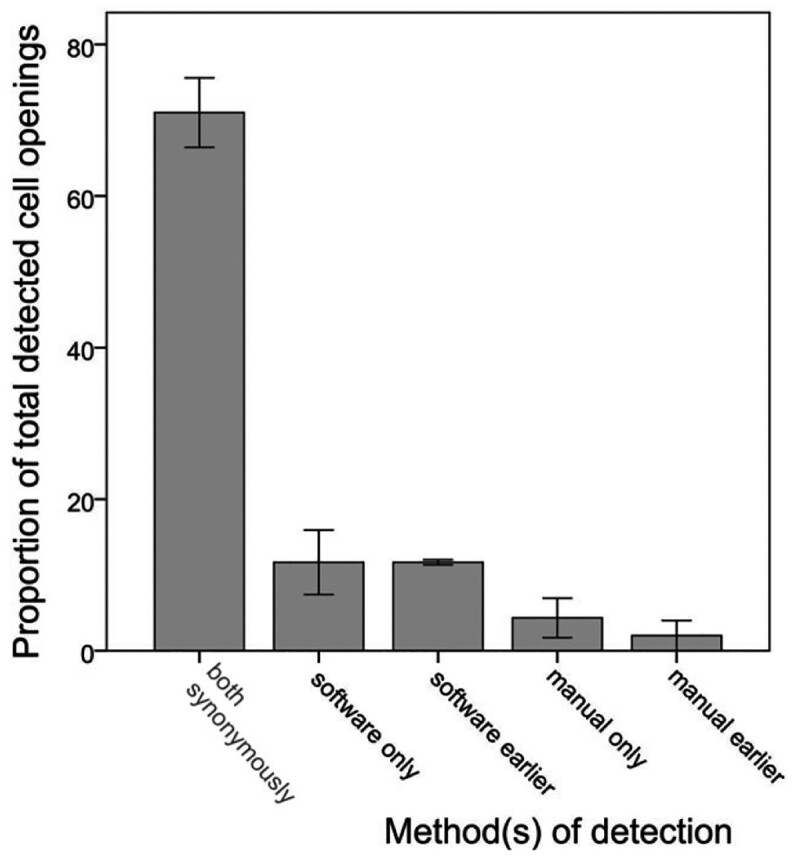
Results of method validation. ‘Both synonymously’ means that both the manual and computer-aided analyses led to the identification to the same individual bee, ‘software only’ means that an uncapping event was detected by the software (and could be confirmed by post-hoc human analysis), while the unaided human observer did not detect any opening. ‘Software earlier’ means that the software enabled detection at an earlier stage, but that the human observer also detected the event (ascribing it to a bee helping in the opening but not initiating it). Each proportion represents the mean with corresponding error bars of one standard deviation.

In these experiments, false positive peaks produced by the algorithm were not recorded, but successfully filtered out by the human controllers. We estimate that three peaks had to be evaluated by the human controller in order to identify the true positive observation of ‘beginning’. Most false positives peaks corresponded to the further continuation of earlier cell opening events, or to instances of trophallactic behaviour (food exchange between worker bees, which involves head movements similar to those used in the feature vector). The total time required for analysing the video output of three experimental rounds, with a sum of 189 infested cells and a recording duration of 231 h, was 86 h for the manual method and 26 h for the computer-assisted method. In 71.0±7.9% of uncappings, the identified initiating bee was identical with both methods (Fig. 7). 11.7±7.4% of uncappings were only identified by the software, against 4.3±4.5% for the manual method. 11.7±0.6% were detected by both methods, but at an earlier stage by the semi-automatic method, and only 2.0±3.5% of events were detected earlier by the manual observer. Overall, manual analysis led to the identification of the correct initiating individual in 77.3% of all detected uncappings, against 94.4% with the semi-automatic method. False positives produced by the software were not analysed here because they could be very quickly eliminated through the human-based control of the software output.

## Discussion

The example chosen here to evaluate our new approach of machine learning is of great economic and ecological importance. Hygienic behaviour is probably the most-used selection trait in breeding for honeybee resistance to parasitic mites, which in turn are seen as the most important causative factor for colony losses in many parts of the world [28, 29, 66]. A great body of literature describes efforts to measure hygienic behaviour or closely related traits such as ‘Varroa Sensitive Hygiene’ or ‘Suppressed Mite Reproduction’ (see e.g. [38, 42, 45, 46, 67–71]). Direct video observation of bees on infested brood is seen as one of the most pertinent ways to measure the trait [46]. It enables the use of the natural removal stimulus (mite infestation/reproduction on brood), as well as the identification of individual worker bees of the ‘hygienic’ phenotype which can then be used for identifying genetic markers. Cell opening events that are later undone through recapping by other worker bees can be detected. However, analyses of video observations have the huge disadvantage of being work-intensive. With the semi-automatic detection method presented here, the amount of human labour required for the analysis of video material from one round of the assay is reduced by a factor of 3.2, from 35.7 to 10.8 h, while the quality of the analysis in terms of the number of detectable events and the correctness of assessments is substantially increased. The total duration of labour, including not only video analysis but also the preparation of the bees and comb, is approximately 75 working hours. Automatization therefore reduces the time investment required for each round of the assay by approximately one-third, from 75 to 50 h. As in one round, bees from up to 30 colonies can be used and the labour required per colony is reduced from 2.5 to 1.7 h. This duration is still high when compared with the freeze- or pin-killed brood assay (approximately 7–20 min/colony; [67]). On the other hand, it is of the same order of magnitude as for other high-precision assays for measuring honeybee resistance traits, such as the ‘Suppressed Mite Reproduction/Varroa-Sensitive Hygiene’-protocol recommended by the Research Network for Sustainable Beekeeping [72].

Of course, measurement of disease resistance is only one example of the application of computer learning to applied and fundamental questions of social insect behavioural research. The approach of [10] extracts behavioural and social features based on position and orientation from tracked honey bees in an observation hive in order to automatically detect one out of four different encounter behaviours. For this task, good results have been achieved by training a classifier based on a single variable, but often more features are needed for a successful classification. In Ref. [15], the effects of neonictonoids on the nursing behaviour and larval development of honey bees are examined. The analysis is based on complex behavioural features pertaining to brood cell visits gathered by a convolutional neural network (CNN), which was trained on within-hive video recordings. For the training of the CNN, data from about 6000 manually classified brood care events were used. Other applications include the analysis of vibroacoustic signals for diagnosing diseases in honeybees [73] and the modelling of ant displacement patterns [18].

Our algorithm is able to incorporate new expert knowledge just by expanding the feature space and automatically learning the corresponding GP hyperparameters by ARD. We note that the GP hyperparameter learning can deal with an arbitrary number of features without overfitting, since ARD assigns a weight close to zero to all uninformative features. It would be interesting to extend the algorithm to the automatic selection of the optimal number of GPs and length of time windows in the final discriminator, which are chosen manually up to now. Here, one could include the final discriminator into a Bayesian model and then use model selection for validation.

Understanding insect societies can inspire solutions to diverse technological and societal processes, from the optimization of production chains to communication strategies within enterprises [4–7]. We hope that the approach taken here can facilitate progress in this direction.

## Supplementary data

Supplementary data are available at *Biology Methods and Protocols* online.

## Data availability

Data from this study are available from the authors upon reasonable request.

## Author contributions

P.B., A.R. and S.T. developed the computer learning algorithm and software, and drafted the manuscript. P.B. analysed the discriminatory power of the features and created Fig. 4. A.R. carried out the numerical experiments for validating the discriminator and created Figs. 5 and 6. J.W. participated in the validation study, co-drafted and edited the manuscript. F.Z. and C.S. designed and carried out the biological experiment as well as the validation study. K.B. conceptualized and supervised the research and edited the manuscript. All authors reviewed the manuscript.

## Funding

This work was supported by the European Union [Fund for Regional Development, grant 85009207, and Seventh Framework Program project SmartBees (Sustainable Management of Resilient Bee Populations; project number 613960)].

*Conflict of interest statement*. P.B., S.T. and A.R. are co-founders of the start-up company Adaptiv Lernende Maschinen GmbH and participated in the present work within the framework of a commercial contract that enabled them to found this company, which exploits the software developed and the know-how gained during the project described here. The other authors declare no conflict of interest.

## Supplementary Material

bpac005_Supplementary_Data
